# The phospholipase A of *Neisseria gonorrhoeae* lyses eukaryotic membranes and is necessary for survival in neutrophils and cervical epithelial cells

**DOI:** 10.1128/mbio.02425-24

**Published:** 2024-09-26

**Authors:** Michael A. Apicella, Jennifer L. Edwards, Margaret R. Ketterer, David S. Weiss, Yuan Zhang, Freda E.-C. Jen, Michael P. Jennings

**Affiliations:** 1 Department of Microbiology and Immunology, The University of Iowa, Iowa City, Iowa, USA; 2 Department of Pediatrics, The Research Institute at Nationwide Children’s Hospital and The Ohio State University, Columbus, Ohio, USA; 3 Institute for Biomedicine and Glycomics, Griffith University, Gold Coast, Queensland, Australia; University of Washington, Seattle, Washington, USA

**Keywords:** *Neisseria gonorrhoeae*, phospholipase A, neutrophils, cervical epithelial cells, hemolysis, gonorrhea

## Abstract

Contact-dependent hemolysins are virulence factors in a number of human pathogens, including *Helicobacter pylori, Rickettsia typhi, Bartonella bacilliformis, Mycobacterium tuberculosis,* entero-invasive *Escherichia coli,* and *Shigella*. Here we demonstrate that *Neisseria gonorrhoeae* produces an outer membrane protein, phospholipase A, that exhibits contact-dependent lytic activity on host cell membranes. This enzyme can lyse human erythrocytes over a 3-day period, whereas a phospholipase A mutant cannot. We demonstrated phospholipase A activity in the parent strain but not in two, independent phospholipase A mutants. A gene for phospholipase A, *pldA* (hereafter referred to as *pla* to avoid confusion with the gene for phospholipase D, *pld*), is present in all sequenced gonococcal strains. Fluid phase, hemolytic activity assays showed that 25 of 29 gonococcal strains tested had hemolytic activity greater than 50% of the positive control. In support of PLA as a gonococcal outer membrane protein, supernatants from 24-, 48-, and 72-h cultures of *N. gonorrhoeae* strain 1291 did not contain hemolysin activity, and a monoclonal antibody specific for gonococcal phospholipase A failed to detect the enzyme in these supernatants. The organism must be viable for lysis to occur, and the inclusion of EDTA in the media removes all activity. Our studies have shown that a phospholipase A mutant has significantly reduced survival in human neutrophils and primary human cervical epithelial cells compared to the parent gonococcal strain after 3 h of incubation. Collectively, our data demonstrate that gonococcal PLA lyses host cell membranes, which is important for intracellular survival.

**IMPORTANCE:**

Intracellular survival is crucial to the success of *Neisseria gonorrhoeae* as a human pathogen. Multiple factors contribute to the intracellular survival of gonococci, including the ability to prohibit apoptosis of the epithelial cell the organism invades and mechanisms to evade host innate defense systems. The role of phospholipase A (PLA), an outer membrane protein, is important as it disrupts the host vacuolar and phagolysosomal membranes, preventing the effective delivery of innate immune factors that normally restrict organism growth within human cells. After cell entry, PLA disrupts the integrity of these host cell membranes, allowing the gonococcus to live free within disrupted vacuoles where it pilfers host cell nutrients that enable its survival and replication. A vaccine or drug that could neutralize PLA activity would disrupt the intracellular survival of the gonococcus.

## INTRODUCTION

*Neisseria gonorrhoeae* (the gonococcus) is an exclusive human pathogen. It is estimated that 1,568,000 new infections occur every year in the United States (1). Globally, *N. gonorrhoeae* infects approximately 87 million people annually. The majority of cases occur in adolescents and young adults.

Since the 1940s, the clinical use, and half-life of an antibiotic for the treatment of gonorrhea has been a little over 15 years, after which it is no longer effective due to the emergence of resistance. Presently, ceftriaxone remains the last first-line monotherapy for gonorrhea in the United States (2). However, with multiple reports of the emergence of ceftriaxone-resistant *N. gonorrhoeae* strains across the globe, ceftriaxone may soon become part of history due to antibiotic resistance. Recent studies from China indicate that this is already occurring (3).

It is established that *N. gonorrhoeae* is an intracellular pathogen (4, 5) within human urethral epithelial cells, cervical epithelial cells (6), and neutrophils (7). Phagocytes are usually effective in killing a wide range of bacteria, but studies show that neutrophils are ineffective in killing the gonococcus (8). Similarly, the organism survives within human genital tract epithelia in both women and men (5, 9). One characteristic of infection is the tendency to frequently see large vacuolar structures containing numbers of gonococci (9–12). In many instances, the vacuolar membranes appear fragmented, suggesting that degradation is occurring and that the gonococcus might be expressing an enzyme capable of modifying phospholipid membranes. To study this, we used a hemolysis assay as a surrogate to test for membrane lysis induced by the gonococcus. These studies led to the observation that the gonococcus exhibits hemolytic activity, which we subsequently showed could be attributed to a phospholipase A, PLA1 (hereafter simply referred to as PLA), a member of the outer membrane PLA superfamily.

PLA is an outer membrane protein (13) and can be classified as a cell contact-dependent membrane lysin. Contact-dependent hemolysins are generally outer membrane proteins that degrade membranes by enzymatic activity. Characteristically, they are not secreted, require contact with the surface they are modifying, and generally act much slower than pore-forming lysins. A review of the literature indicated that many bacterial species have contact-dependent hemolysins, including the human pathogens *Campylobacter coli* (14), *Rickettsia prowazekii* (15), *Vibrio vulnificus* (16), *Treponema lecithinolyticum* sp. (17), *Campylobacter jejuni* (18), and *Pseudomonas aeruginosa* (19). Each of these bacteria has PLA analogs that are contact-dependent lysins that are capable of modifying host membranes and that display approximately 29% to 65% identity to gonococcal PLA at the protein level (see Table S1). Prior analyses of gonococcal PLA have focused on a potential role for this protein as an autolysin (13, 20). In this paper, we provide evidence that gonococcal PLA also is a contact-dependent (hemo)lysin, and loss of PLA activity is responsible for significantly decreased gonococcal survival in both neutrophils and primary human cervical epithelial cells.

## MATERIAL AND METHODS

### Bacteria

#### Bacterial strains

*N. gonorrhoeae* strains tested included 1291 (21, 22) and FA1090 (23). These bacteria were originally isolated from patients with culture-documented gonococcal disease and are commonly used to study *N. gonorrhoeae* pathogenesis. Strain 1291 is a male urethral isolate, whereas strain FA1090 is a genital isolate from a woman with disseminated infection. A panel of *N. gonorrhoeae* strains (see Table 1) isolated from various sites of infection and causing different disease outcomes (e.g., disseminated infection, pelvic inflammatory disease, and uncomplicated gonorrhea) was also tested. The FA1090*hylA* mutant was a gift from Dr. Hank Seifert of Northwestern University, Chicago, IL. The 1291*pld* mutant is previously described (24). Bacteria were cultured overnight (37°C, 5% CO_2_) on GC medium base (Difco, Sparks, MD) supplemented with 1% IsoVitaleX (Becton Dickinson, Franklin Lakes, NJ) agar (GCA) plates and enumerated spectrophotometrically before their use in the experiments described herein.

**TABLE 1:** Analysis of lytic activity among *N. gonorrhoeae* strains

Gonococcal strain	Hemolytic index^*a*^
Disseminated infection	
DGI-3	84.54
DGI-11	94.69
DGI-18	95.26
DGI-19	101.60
DGI-29	93.80
DGI 33	70.80
DGI 34	78.31
DGI 36	100.92
DGI 37	10.89
DGI 41	25.24
DGI 42	71.97
DGI 44	78.22
FA-19	99.37
FA-1090	81.36
Pelvic inflammatory disease	
PID-8	69.10
PID-18	87.57
PID-24-1	64.72
PID-302	102.75
PID-305	79.04
PID-332	110.37
PID-335	66.22
Uncomplicated disease or unknown source	
NJ-4	98.54
NJ-18	117.43
NJ-35	84.65
NJ-39	44.30
538112	118.54
179	79.21
398081	20.20
1291	90.08

^a^ The hemolytic index was calculated as a percentage of values obtained for the 100% lysis control. Gray shaded fields denote those strains exhibiting lytic activity below a 50% threshold.

For *N. gonorrhoeae* broth cultures used (as noted) in neutrophil infection studies, overnight GCA cultures were harvested into 5 mL GC broth supplemented with 1% IsoVitaleX (GCB) and then incubated (37°C, with shaking at 200 rpm) to adapt to growth in liquid. Bacteria were collected (4,000 × *g*) after 2.5 h to 3 h of growth in GCB and resuspended in 1.5–2 mL Hank’s Balanced Salt Solution (HBSS) with Ca^2+^ and Mg^2+^ (HBSS++; Gibco, Grand Island, NY). Cultures were adjusted to a final concentration of 2 × 10^8^ colony forming unit (CFU)/mL and incubated (37°C, no rotation) for 20–30 min to allow pili to re-form.

#### Construction of pla knockout mutants in N. gonorrhoeae

A 39 bp-truncated *pldA* (hereafter referred to as *pla* to avoid confusion with the gene for phospholipase D, *pld*) gene (NGO1492 in FA1090, UniProt accession #Q5F6Q7, GenBank accession #AAW90130) of *N. gonorrhoeae* strain 1291 (1291 genome from reference 25), including 200 bp downstream of *pla,* was amplified with primers 1492truncF and 1492truncR (Table S2) and cloned into pT7blue (Novagen, Madison, WI). The resulting plasmid was named pT7blue*plA*. The tetracycline resistance cassette was obtained from pGEM-TetMA (26) by digestion with *Hinc*II and ligated into the *EcoR*V site of pT7blue*pl*A to create the plasmid pT7blue*plA::tetM* (see Fig. S1). pT7blue*plA::tetM* was then linearized with *Not*I and transformed into *N. gonorrhoeae* strain 1291. The recombinant strain (1291*pla::tet*) was selected by growth on GCA containing 2.5 µg/mL tetracycline. The presence of the tetracycline cassette was confirmed by PCR using the primer pair NGO1492F and NGO1492R (Table S2) and by sequencing. FA1090*pla::tet* was generated by transforming wild-type FA1090 with *pla::tet* DNA, amplified from 1291*pla::tet* using PLA primer 1 and PLA primer 2 (Table S2). Selection of FA1090*pla::tet* transformants and mutant verification were performed as described for the 1291*pla::tet* mutant.

The 1291*pla*::erm mutant was generated in a similar manner. The erythromycin (erm) resistance cassette was obtained by digestion of pCatErm (kindly provided by Dr. H. Seifert) (27) with *Sph*I *and Sca*I. Full-length *pla* was amplified from strain 1291 using the primer pair PLA-414 and PLA+508, which was then cloned into pNEB193 (New England Biolabs, Ipswich, MA) to generate pNEB*pla*. pNEB*pla* was digested with *EcoR*V and *Hpy166*II to which the erythromycin resistance cassette was blunt-end-ligated with the removal of a 420 bp fragment of *pla* to form pNEB*pla-erm*. pNEB*pla-erm* was linearized with *pvu*II, and used to transform strain 1291, after which transformants were selected on GCA supplemented with 50 µg/mL erythromycin. 1291*pla*::erm mutants were verified by PCR using the primers PLA/ermC Up R and PLA/ermC Down F and sequencing across the entirety of *pla*.

Enzyme-linked immunosorbent assay analyses confirmed comparative expression levels for pilus, Opa, lipooligosaccharide, and the conserved H.8 epitope between wild-type 1291 and its *pla* mutants (Fig. S2).

#### Expression of N. gonorrhoeae pla in Escherichia coli

*E. coli* strain, EC251 (also known as MG1655) (28), was transformed to express full-length, *N. gonorrhoeae* 1291 *pla*. EC251 does not produce a PLA protein with hemolytic activity (see Fig. 2). Gonococcal *pla* plus 250 bp of upstream DNA was amplified by PCR using primers P2936 and P2937 (Integrated DNA Technologies; Coralville, IA) (Table S2). The resulting 1,444 bp product was introduced into *Hind*III-digested pACYC184 (29) by isothermal assembly using NEBuilder HiFi Assembly Master Mix from New England Biolabs. Plasmid inserts were verified by DNA sequencing and used to transform chemically competent, EC251 *E. coli*. EC251/pACYC184::*pla* and the empty-vector control strain EC251/pACYC184 were grown (30°C) on Luria Bertani (LB) agar supplemented with 30 µg/mL chloramphenicol and 2% human erythrocytes to test for hemolytic activity, as described below.

### Cell culture

Neutrophils were isolated from human blood as described previously (11, 30). In brief, heparinized blood was drawn from healthy volunteers according to a protocol approved by the Institutional Review Board at The University of Iowa. Neutrophils were isolated by dextran sedimentation and Hypaque-Ficoll (GE Healthcare, Chicago, IL) density gradient separation. Residual erythrocytes were removed by hypotonic lysis, after which neutrophils were resuspended in HBSS without Ca^2+^ and Mg^2+^. Isolation yielded neutrophil cultures of >99% purity and that were >95% viable as measured by trypan blue exclusion.

Primary human cervical epithelial (Pex) cells were procured from surgical cervical tissue and maintained in a defined keratinocyte serum-free medium (Gibco), as previously described (6). Deidentified, healthy, cervical tissues were obtained from premenopausal women and were provided by the Human Tissue Resource Network/Cooperative Human Tissue Network (Columbus, OH). The use of these tissues does not constitute human subjects research, as determined by the Institutional Review Board.

### Hemolytic activity assays

#### Erythrocyte substrate

Fresh whole human blood was collected with EDTA as the anti-coagulant. Aliquots of blood were washed twice in cold RPMI-1640 (Gibco). For solid phase assays, washed erythrocytes were added at a final 2% vol to GCA or LB agar; 10 mL of agar was poured into each Petri plate. For liquid phase assays, the washed erythrocytes were suspended to a final volume of 4% in RPMI-1640 (with glutamine but without phenol red) supplemented with 0.8 mg/mL glucose and 1% IsoVitaleX.

#### Solid phase assay

Bacteria from frozen stock cultures were streaked onto blood agar plates and incubated (37°C, 5% CO_2_) for the noted, experimentally-determined, interval, typically 72 h. Colony growth and the area of erythrocyte clearing around the colonies, or streaks, were photographed to record hemolysis.

#### Liquid phase assay

Overnight GCA cultures of *N. gonorrhoeae* were suspended in RPMI-glucose-IsoVitaleX medium, and the culture density was adjusted to an OD_600_ = 2.0. Equal volumes of washed erythrocytes and bacteria were then combined to initiate the reaction. In this regard, four replicate samples were tested per assay with each sample containing a mixture of gonococci at an OD_600_ = 1.0 and washed erythrocytes at 2%. Negative control mixtures were made with media alone (no bacteria) and washed erythrocytes; positive control mixtures consisted of media and washed erythrocytes lysed in sterile water. Following a 72 h incubation [37°C with gentle (70 rpm) rotation], residual erythrocytes were pelleted (10 min, 1,400 × *g*) from each reaction mixture. Hemolysis was then evaluated by measuring the A_450_ of triplicate 100 µL aliquots of supernatant from each sample. The hemolytic index was calculated for each specimen as the average A_450_ of the specimen/average A_450_ of the positive control. Data shown were derived from at least three separate assays.

### PLA monoclonal antibody production and verification

The vector, pET-15b (GenScript Biotech, Piscataway, NJ) was used to express the N-terminal, 136 amino acids of truncated PLA in the *E. coli* strain, BL21 DE3. After induced expression with IPTG, the recombinant, truncated PLA protein was purified in 8 M urea, 0.1 M glycine, 20 mM Tris, pH 8.3, and 2 mM EDTA after which it was buffer-exchanged to 1 M urea in PBS. BALB/c mice were injected subcutaneously with 25 µg of purified PLA mixed with Freund’s complete (day 0) or incomplete (days 21 and 28) adjuvant (Sigma-Aldrich, St. Louis, MO). Test bleeds were collected on day 42. The mouse with the best reactivity to purified PLA and PLA in *N. gonorrhoeae* (Fig. S3) was sent to WEHI (Walter and Eliza Hall Institute of Medical Research, Parkville, VIC AUS) for monoclonal antibody (mAb) production and hybridoma generation. Hybridoma mAbs were screened by western blotting using purified PLA and *N. gonorrhoeae* 1291 wild type and 1291*pla* mutant cell lysates (Fig. S4). Antibodies then were purified, and antibody isotypes were classified by WEHI.

### Phospholipase A activity assay

Gonococcal PLA activity was measured using the fluorogenic PLA_1_ and PLA_2_ substrate bis-BODIPY FL C11-PC (Invitrogen, Molecular Probes; Eugene, OR) in a microplate assay, as described by the manufacturer with slight modification. In brief, *N. gonorrhoeae* strains 1291, 1291*pla::tet*, and 1291*pla::erm* were harvested from overnight GCA cultures and enumerated spectrophotometrically. Gonococci were suspended in assay buffer (50 mM Tris-HCL, 100 mM NaCl, 1 mM CaCl_2_) at a concentration of 10^9^ bacteria/mL and then lysed by sequentially pulling them through 22-, 23-, 25-, and 26-gauge needle syringes. PLA_2_ from bee venom (Sigma-Aldrich) was diluted in an assay buffer to a concentration of 2 U/mL (positive control). Liposomes were generated by mixing equal volumes of 1 mM bis-BODIPY FL C11-PC, 10 mM dioleoyl phosphatidylcholine, and 10 mM dioleoyl phosphatidylglycerol (both from Avanti Polar Lipids, Alabaster, AL) in assay buffer. To initiate the reactions, 50 µL of enzyme (bacterial lysate or bee venom) or assay buffer (negative, no lysis, control) was added to the desired wells of a 96-well microtiter plate, after which 50 µL of the liposomal mixture was added to each well. Fluorescence (488/530 nm), indicative of PLA activity, was then recorded every 5 min for 1 h using a Synergy HT Multi-mode Microplate Reader (BioTek Instruments, Winooski, VT, USA). Assays were performed in triplicate on three separate occasions. Data were adjusted for background (blank wells), and the statistical significance of the data obtained was determined using a Student’s *t*-test.

### Isolation of *E. coli* membranes

*E. coli* strain EC251/pACYC184::*pla* and the empty-vector control strain, EC251/pACYC184, were grown in 500 mL LB-chloramphenicol broth at 37°C, 210 rpm to OD_600_ = 1.0. Cultures were chilled on ice, then cells were harvested by centrifugation at 5,000 × *g* for 8 min. Cell pellets were stored at −80°C until use. Pellets were thawed, suspended in 3.5 mL buffer (50 mM Tris, 150 mM NaCl, 10 mM CaCl_2_, pH 7.4), and lysed by sonication. Unbroken cells and large debris were removed by two successive centrifugations (12,000 × *g*, 4°C, 10 min). The supernatant from the second centrifugation was then centrifuged at 100,000 × *g* (4°C, 1.5 h) to pellet membranes. The membrane pellet was taken up in a 1.5 mL cold buffer. Protein concentrations were determined using a BCA Protein assay kit (Pierce, Rockford, IL) using bovine serum albumin as a standard. Initial concentrations were about 25 mg/mL. Samples were adjusted to 10 mg/mL with buffer and stored at −20°C until use.

### *N. gonorrhoeae* association, invasion, and survival assays

Neutrophils (2 × 10^6^) were seeded to 13 mm plastic coverslips (Sarstedt Inc., Newton, NC) in 24-well plates. Adherence was aided by centrifugation (10°C, 5 min, 1,000 rpm), followed by a 30 min incubation (5% CO_2_) at 37°C. The plates with the adherent neutrophils were then held for a minimum of 15 min at 4°C to allow bacteria preparation. Piliated, *N. gonorrhoeae* GCB cultures (see “*Bacteria*” above) were diluted to a final concentration of 1 × 10^8^ CFUs/mL in 1 mL warm RPMI supplemented with 1% IsoVitaleX and 1% heat-inactivated pooled human serum (hiPHS). Neutrophils were spinoculated (10°C, 5 min, 1,600 rpm) with gonococci at a multiplicity of infection (MOI) of 5, after which the medium was immediately replaced with 500 µL of warm RPMI-IsoVitaleX-hiPHS medium, and the plates were incubated (37°C, 2 min) to allow synchronous phagocytosis of the bacteria by the adherent neutrophils. The coverslips were then washed thrice with Dulbecco’s PBS with Ca^2+^ and Mg^2+^ (PBS++) to remove loose bacteria and non-adherent neutrophils (defined as phagocytosis time = 0). The final wash was replaced with 500 µL warm RPMI-IsoVitaleX-hiPHS medium, and the cells were incubated (37°C, 5% CO_2_) for select times, as noted. At each time-point, including t = 0, infected neutrophils were rinsed thrice with PBS++ and lysed by incubation (37°C, 10 min) in warm, 1% saponin to release intracellular bacteria. Viable bacteria were enumerated by plating serial dilutions of the neutrophil cell lysates onto GCA (triplicate replicates) and counting CFUs after overnight incubation (37°C, 5% CO_2_). Assays were performed on at least three separate occasions, and significant differences in the calculated percentage of viable wild type vs mutant gonococci for each assay were determined using a non-parametric ANOVA.

Pex cell monolayers were infected with *N. gonorrhoeae* strains 1291 or 1291*pla::tet* at an MOI of 100. Quantitative survival assays were then performed as we have described (31). Briefly, Pex cells were infected with gonococci for 90 min after which gentamicin was then omitted from (association assays) or added to (invasion and intracellular survival assays) infected cell monolayers to kill extracellular, cell-associated bacteria. Pex cell monolayers were subsequently rinsed and lysed or they were subject to a second 1- or 3-h incubation in an antibiotic-free medium before cell lysis (intracellular survival assays). For all assays, serial dilutions of the cell lysates were plated to enumerate viable CFUs. The percent association, invasion, or survival was determined as a function of the original inoculum and the number of colonies formed with subsequent plating of the cellular lysate. A non-parametric analysis of variance was used to determine the statistical significance of the calculated mean percent association, invasion, or survival for each assay. The data shown are the results of three assays performed in triplicate.

### Live/dead staining to assess *N. gonorrhoeae* viability in neutrophils

Piliated, *N. gonorrhoeae* were grown in GCB, as above. Culture density was adjusted to 2 × 10^8^ CFU/mL in warm RPMI-IsoVitaleX-hiPHS medium, which was then mixed with an equal volume (0.5 mL) of freshly isolated neutrophils (2 × 10^7^) in 4-(2-hydroxyethyl)-1-piperazineethanesulfonic acid (HEPES)/HBSS (without Ca^2+^ or Mg^2+^) to yield an MOI of 10. This infection mixture was incubated (37°C, 20 min) with gentle tumbling to allow the gonococcal-neutrophil interaction to occur. After this initial attachment period, bacterial and host cells were pelleted (5 min, 1,000 rpm), and the supernatants (containing loose bacteria not associated with neutrophils) were discarded. The cell pellets were resuspended in the original volume (1.5 mL) of buffer; this represented time = 0. At the noted times, 100 µL aliquots of each cell mixture were removed for labeling with LIVE/DEAD Fixable Cell Stain (Invitrogen, Waltham, MA), per the manufacturer’s instructions. In brief, the PMN+bacteria mixtures from each time-point were incubated (15 min, room temperature, in the dark) with the live/dead stain after which they were resuspended in 100 uL 4% paraformaldehyde before transferring to poly-L-lysine-coated microscope slides and viewed with the Olympus BX-61 microscope housed in the Central Microscopy Research Facility at the University of Iowa.

### Transmission electron microscopy

PMN-gonococcal mixtures were set up for the live/dead stain (above), and aliquots were removed at specified times following the initial incubation to allow attachment. These aliquots were fixed in glutaraldehyde/0.1 M sodium cacodylate buffer (pH 7.4) to a final 2.5% and stored at 4°C. At the time of processing for microscopy, a portion of each sample was immobilized in a plug of 1% agarose/PBS. This plug was fixed again in 2.5% glutaraldehyde, post-fixed with 1% osmium tetroxide for 1 h, and then rinsed in 0.1 M sodium cacodylate buffer. Following serial alcohol dehydration, the samples were embedded in Epon 12 (Ted Pella, Redding, CA). Ultrathin sections (70 nm) were post-stained with uranyl acetate and lead citrate. Samples were examined with a Hitachi HT-7800 transmission electron microscope (Tokyo, Japan) housed at the Central Microscopy Facility at the University of Iowa.

## RESULTS

### *N. gonorrhoeae* exhibit hemolytic activity

**Fig 1:**
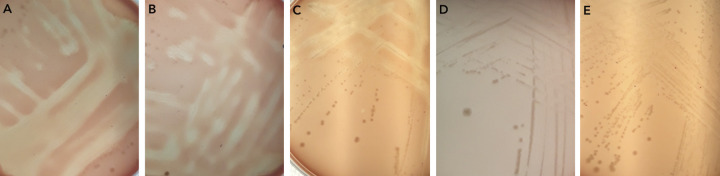
Contact-dependent gonococcal hemolytic activity can be attributed to phospholipase A***.*** An area of clearing on the blood agar plates is indicative of hemolysis and was observed for strains 1291 (**A**), 24-1 (**B**), and FA1090 (**C**) on GCA-blood plates by 72 h of incubation. In contrast, *N. gonorrhoeae* 1291*pla* (**D**) and FA1090*pla* (**E**) did not hemolyze human erythrocytes. In separate studies, we analyzed a gonococcal phospholipase D mutant and an HylA mutant (data not shown). These two mutant strains were able to hemolyze erythrocytes as well as the parent strains, indicating that PLA was the required enzyme.

In our past electron microscopy studies of *N. gonorrhoeae* within human cells, one characteristic that has stood out is the amount of vacuolar disruption and fusion that occurs (8, 9, 12). Given that vacuolar structures are rich in phospholipids, we initiated a search for an enzyme capable of lysing phospholipid membranes by examining colonies of *N. gonorrhoeae* strain 1291 for evidence of zones of hemolysis surrounding colonies on GCA plates supplemented with 2% human erythrocytes. After 24 h of incubation, we detected what appeared to be very narrow zones of hemolysis around the colonies. This zone of hemolysis expanded over the next 48 h. Images in Fig. 1A through C show zones of hemolysis either around colonies or streaks of colonies of *N. gonorrhoeae* strains 1291, 24.1, and FA1090 at 72 h after inoculation. These studies indicated that the gonococcus encodes an enzyme, or a series of enzymes, that can degrade phospholipid-containing membranes. Given the relatively delayed nature of the lysis observed, we decided the responsible enzyme was most probably a cell-contact lysin.

As cellular membranes are phospholipid-rich structures, a phospholipase produced by the gonococcus could be a factor in disrupting phagolysosomes and/or endocytic vacuoles. The gonococcal genome contains homologs of phospholipase A (*pla*), and phospholipase D (*pld*), as well as an alpha-hemolysin homolog (*hylA*). To determine whether any of these gene products could be the potential lysin in our studies, we mutated each of these genes in *N. gonorrhoeae* strain 1291 and *N. gonorrhoeae* FA1090 and then analyzed the ability of these mutants to lyse human erythrocytes in our solid phase assay. Only the *N. gonorrhoeae* 1291*pla* and FA1090*pla* mutants were unable to lyse erythrocytes (Fig. 1D and E), indicating that the PLA protein was responsible for the hemolysis.

**Fig 2:**
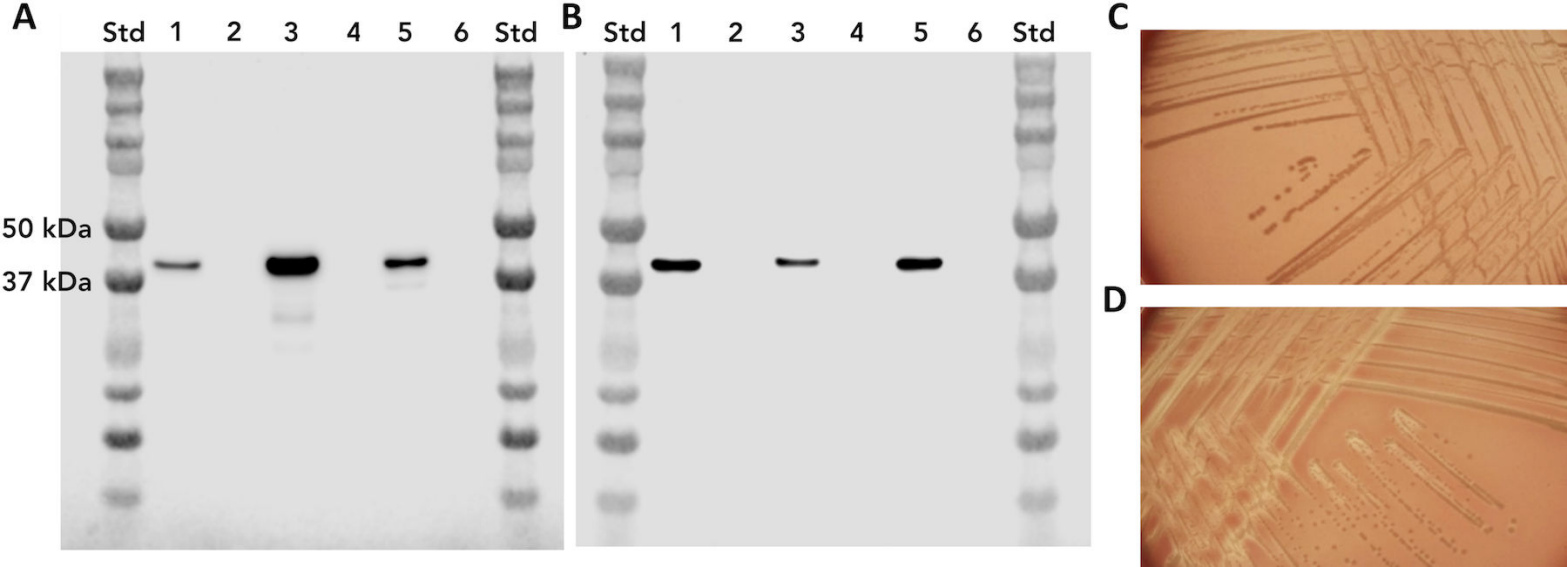
Western blot demonstrating PLA expression in *N*. *gonorrhoeae* strain 1291 and *E. coli* and hemolysin activity in PLA transformed *E. coli*. Panel A shows a western blot probed with anti-PLA monoclonal antibody 1E8. Lanes: 1—1291WT lysate; 2—1291*pla::tet* lysate; 3*—E. coli* 6516:pACYC184::GC*pla* lysate; 4*—E. coli* 6514:pACYC184 lysate; 5—4 µg membrane prep from *E. coli* 6516:pACYC184::GC*pla*, 6—4 µg membrane prep from *E. coli* 6514:pACYC184. (**B**) Lanes 1 and 2—same as Panel A; lane 3—2 µg membrane prep from 1291WT; 4—2 µg membrane prep from 1291*pla::tet*; 5—4 µg membrane prep from *E. coli* 6516:pACYC184::GC*pla*; 6—4 µg membrane prep from *E. coli* 6514:pACYC184. These data demonstrate that the gonococcal PLA is expressed in both *N. gonorrhoeae* as well as the transformed *E. coli* and that the protein was present in both bacterial cell membranes. Panel C demonstrates that *E. coli* with the empty plasmid, pACYC184, has no zone of hemolysis around the colonies, whereas *E. coli* transformed with a plasmid expressing the gonococcal *pla* shows zones of hemolysis around the colonies (**D**).

Attempts to complement the *pla* mutant in *N. gonorrhoeae* were unsuccessful because of our inability to obtain *pla* in high copy number *E. coli* plasmids without a mutated sequence, suggesting that the intact gene forms a lethal product in *E. coli* at high copy number. However, we were able to clone *pla* into the low copy number plasmid, pACYC184, and introduce this into *E. coli*. Figure 2A and B show western blots, probed using the anti-PLA monoclonal antibody 1E8 (Fig. S3 and S4), that demonstrate the presence of the protein as a 48 kDa protein in *N. gonorrhoeae* strain 1291 and its absence in strain 1291*pla* (Fig. 2A). In addition, this image shows the expression of gonococcal PLA in *E. coli* as well as its presence in the membrane fraction of gonococcal *pla*-transformed *E. coli*. Fig. 2B demonstrates the presence of the protein in the membrane fraction of the gonococcus. The *E. coli* transformant also demonstrated hemolytic activity, which was not observed in the host strain (Fig. 2C and D).

**Fig 3:**
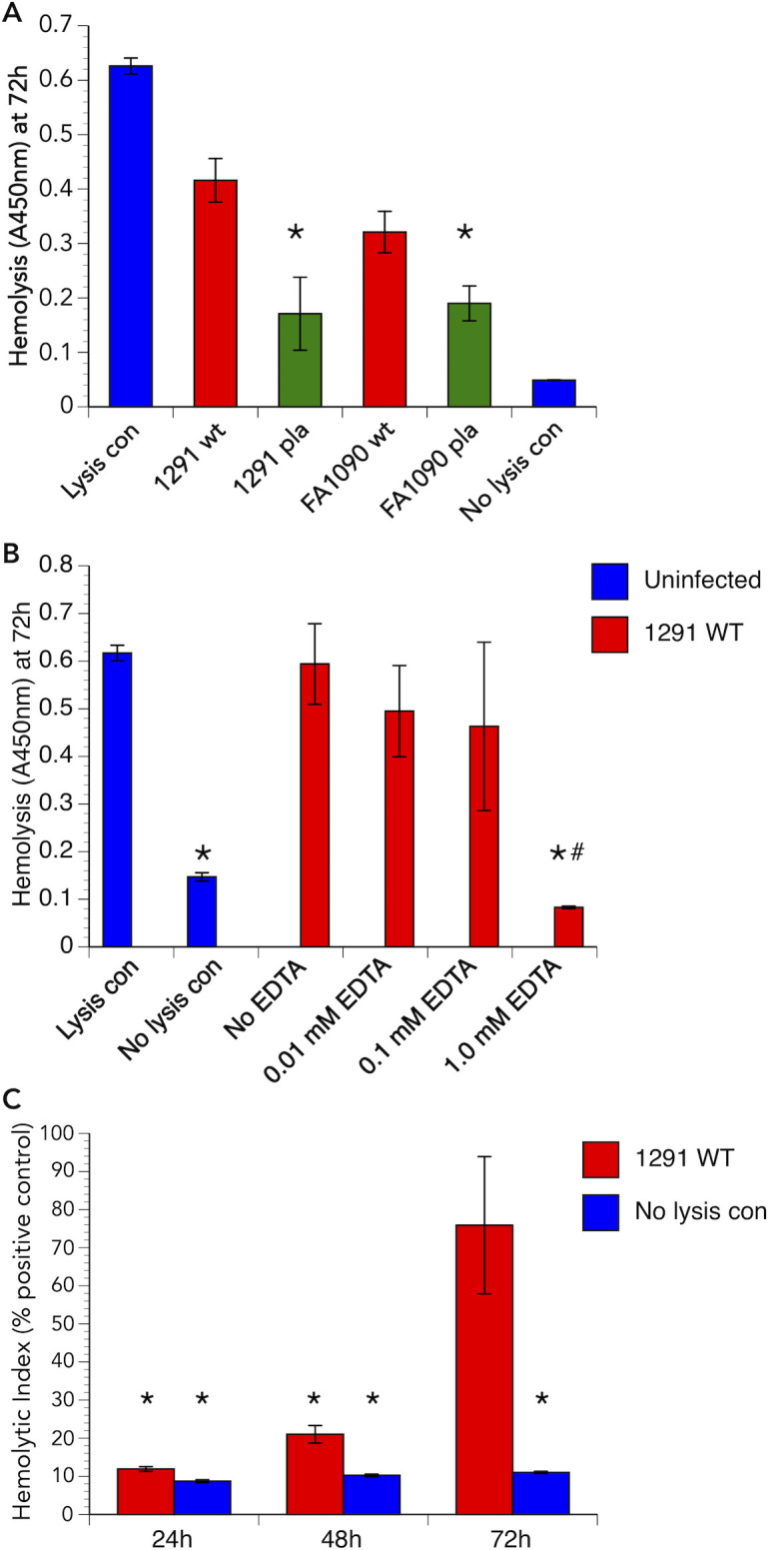
Solution-based assay for the detection of *N. gonorrhoeae* PLA activity. (**A**) Solution-based hemolysis assay using *N. gonorrhoeae* strains 1291, 1291*pla,* FA1090, and FA1090*pla* (noted on the x-axis). As can be seen, strains 1291 and FA1090 hemolyzed the erythrocytes, whereas the respective phospholipase A mutants from each strain did not. *P ≤ 0.0198 vs the respective wild-type strain. (**B**) Hemolytic activity for strain 1291 exhibits a dose-dependent decrease in the presence of EDTA, which disrupts homodimer formation, and thereby the activity, of PLAs. *P ≤ 0.0001 vs lysis (positive) control; # *P* ≤ 0.0018 vs no EDTA. (**C**) Time course experiment showing hemolysis over 72 h in the solution-based assay. Hemolysis developed over time in the gonococcus, which is consistent with data obtained using the solid phase assay. *P ≤ 0.0059 vs positive lysis control.

To facilitate our studies, we developed a solution-based assay. This approach allows for the validation of the solid phase assay and provides semi-quantitative results. Gonococcal hemolytic activity was compared to a 100% lysis control. Figure 3A shows the results of such a study. Both strains 1291 and FA1090 showed hemolysis in the assay, which was significantly (*P* ≤ 0.0198) reduced in their respective *pla* mutants. Moreover, the activity observed for the 1291*pla* mutant did not differ significantly (*P* = 0.1225) from the no lysis control.

Bacterial PLAs are homodimers, requiring a Ca^2+^ ion to non-covalently link two identical peptides for activity (32). To test this with gonococcal PLA, we performed the fluid phase assay in the presence and absence of EDTA, a cation chelator. In the presence of EDTA, a dose-dependent decrease in lytic activity was observed for wild-type 1291 with increasing concentrations of EDTA (Fig. 3B). Time course experiments at 24, 48, and 72 h after initiation of contact (Fig. 3C) demonstrated an increase in hemolytic activity from approximately 10% at 24 h to 20% at 48 h and greater than 75% by 72 h. This mimics the kinetics of the solid phase assay and provides further evidence that gonococcal PLA is a contact-dependent lysin. Using the anti-PLA monoclonal antibody 1E8, we demonstrated that PLA was absent from the supernatants at each of these time points, further confirming the cell-contact-dependent nature of the PLA lysin (data not shown).

As mentioned, based on NCBI sequence analysis, PLA is present in the majority of gonococcal strains sequenced to date. To test for lytic activity across gonococcal strains, we analyzed 29 strains that differed in their clinical situation (i.e., pelvic inflammatory disease, disseminated gonococcal infection, urethritis, and cervicitis). In the fluid phase assay, all but four strains showed signs of hemolytic activity greater than 50% of the control by 72 h (Table 1). The remaining strains exhibited low levels (≤44.3% vs the control) of hemolysis. Sequence analysis of *pla* in two of these strains (NJ39 and 398081) demonstrated that each was identical to that of the 1291 and FA1090 *pla* sequences, suggesting that another factor may be involved in their reduced lytic activity. Interestingly, these strains did react with monoclonal antibody 1E8 in western blot. The reason for their reduced lytic activity is unknown and may relate to regulatory issues associated with the enzyme.

Based on the similarities between hemolysis seen with *C. jejuni* (18)*,* which produces cell-contact-dependent PLA (hemo)lysins, we reasoned that *N. gonorrhoeae* lytic activity necessitated cell contact and viable bacteria. To determine whether this hypothesis was correct, we treated the bacterial suspension with 5 µg/mL of chloramphenicol prior to incubation with human erythrocytes. No lysis was seen in these experiments, indicating that, in addition to cell contact, viable bacteria were necessary for lysis to occur (data not shown).

### Gonococcal PLA exhibits phospholipase A activity

**Fig 4:**
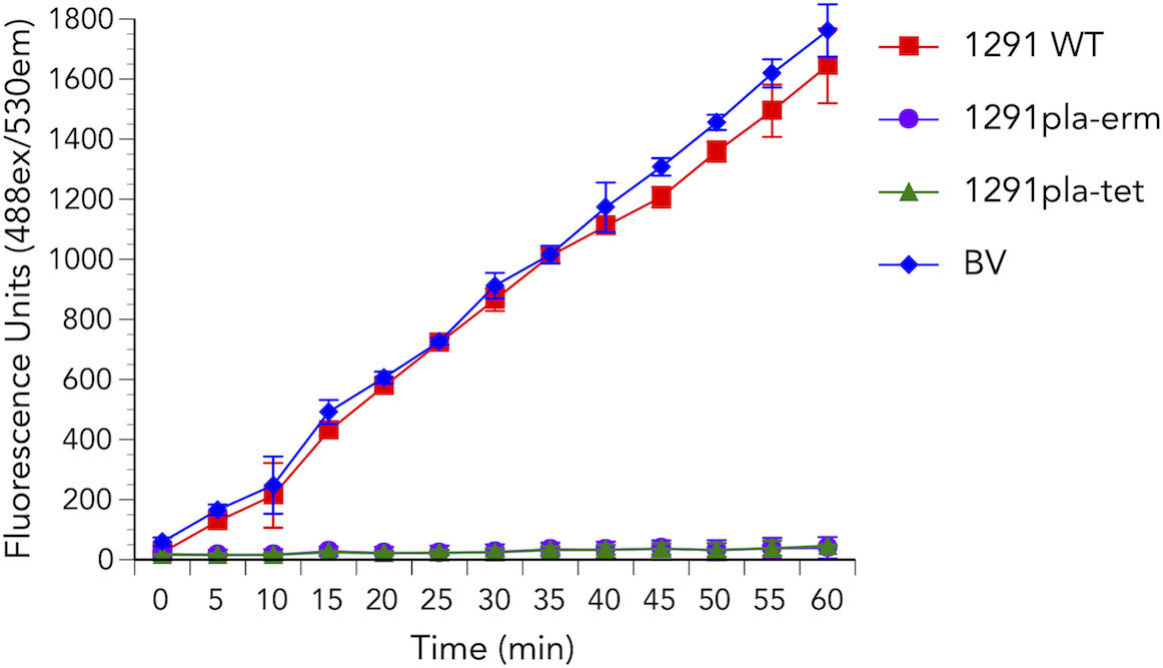
PLA activity in *N. gonorrhoeae*. Gonococcal PLA activity was measured using the fluorogenic PLA_1_ and PLA_2_ substrate bis-BODIPY FL C11-PC in a microplate assay, as described in the Materials and Methods. PLA_2_ from bee venom (BV) was used as a positive assay control. Fluorescence (488/530 nm, y-axis), indicative of PLA activity, was recorded every 5 min for 1 h (x-axis). Assays were performed in triplicate on three separate occasions. A statistically significant (*P* ≤ 0.0001) difference in PLA activity was observed for both *pla* mutants when compared to the wild-type strain at all times commencing at 15 min post-reaction initiation (0 times). Fluorescence recorded for 1291 wild type was not significantly (*P* ≥ 0.1055) different from the positive control at most time points (*P* ≤ 0.0336 at 45 min, *P* ≤ 0.0313 at 50 min).

To confirm that the gonococcal PLA candidate has the phospholipase A activity theoretically assigned to it, we tested wild-type *N. gonorrhoeae* strain 1291 and two independent 1291*pla* mutants, 1291*pla::erm* and 1291*pla::tet,* for their phospholipase A activity using liposomes composed of the fluorogenic PLA substrate, bis-BODIPY FL C11-PC, dioleoyl phosphatidylcholine, and dioleoyl phosphatidylglycerol. PLA from bee venom was used as a positive control, and data were adjusted relative to the liposome-only negative control. Figure 4 shows that gonococcal strain 1291 has PLA activity, which was not observed for the 1291*pla::erm* or 1291*pla::tet* mutants. These data confirmed that NGO1492 (i.e., *pla*) exhibits phospholipase A activity.

### Phospholipase A activity contributes to gonococcal intracellular survival during infection

**Fig 5:**
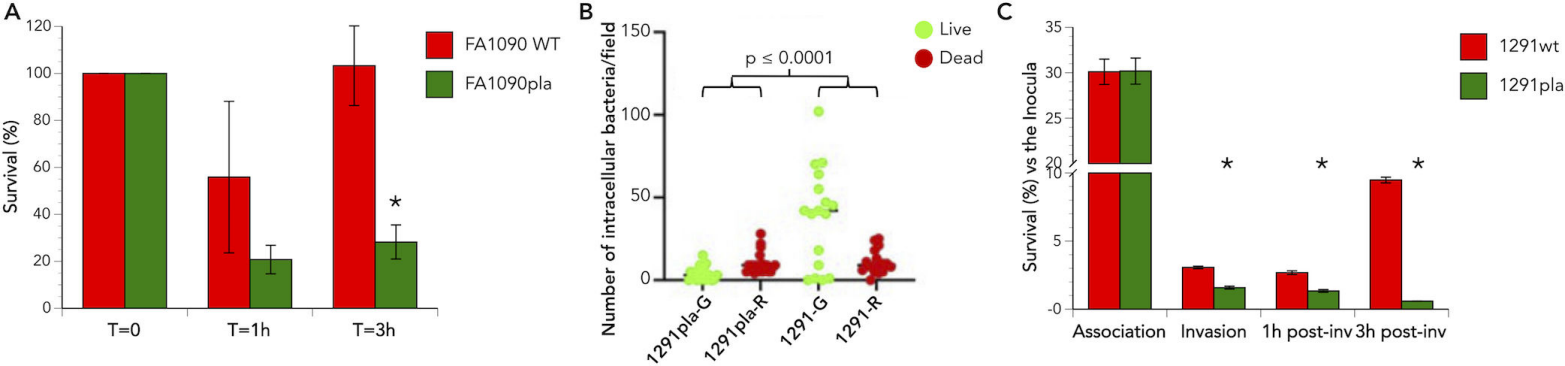
PLA contributes to gonococcal survival during infection of primary human cells. (**A**) Neutrophils were synchronously infected with FA1090 or FA1090*pla* gonococci, as described in the text, after which the cells were lysed and plated to enumerate viable colonies (T = 0) or the infection was allowed to proceed. At 1 h after infection (T = 1 h) of human neutrophils, both the wild-type strain FA1090 and the FA1090*pla* mutant showed a decrease in survival, which was not significantly different. By 3 h after infection (T = 3 h), the FA1090 wild-type strain showed a significant recovery in survival (indicative of intracellular replication) when compared to the mutant strain FA1090*pla*, which failed to recover. *P ≤ 0.0004 vs wild type. (**B**) Live/dead staining shows the results of counting individual bacteria within neutrophils in 20, random, 60× fields for the 1291*pla* mutant and 1291 wild type at 3 h post-infection. Viable bacteria fluoresce green (**G**), while the dead organism fluoresce red (**R**). This study indicates that the PLA mutation rendered the gonococcus susceptible to human PMN killing. (**C**) Quantitative association, invasion, and survival assays were performed following Pex cell infection with *N. gonorrhoeae* strains 1291 or 1291*pla::tet*, as described in the Materials and Methods. The data shown are the mean (variance) of three assays performed in triplicate. Whereas no differences were observed in the ability of wild type and 1291*pla* mutant bacteria to associate with Pex cells, 1291*pla* mutant bacteria were less invasive and exhibited a significant survival defect by 3 h post-invasion vs the parental wild-type strain. *P ≤ 0.0005 vs wild type.

**Fig 6:**
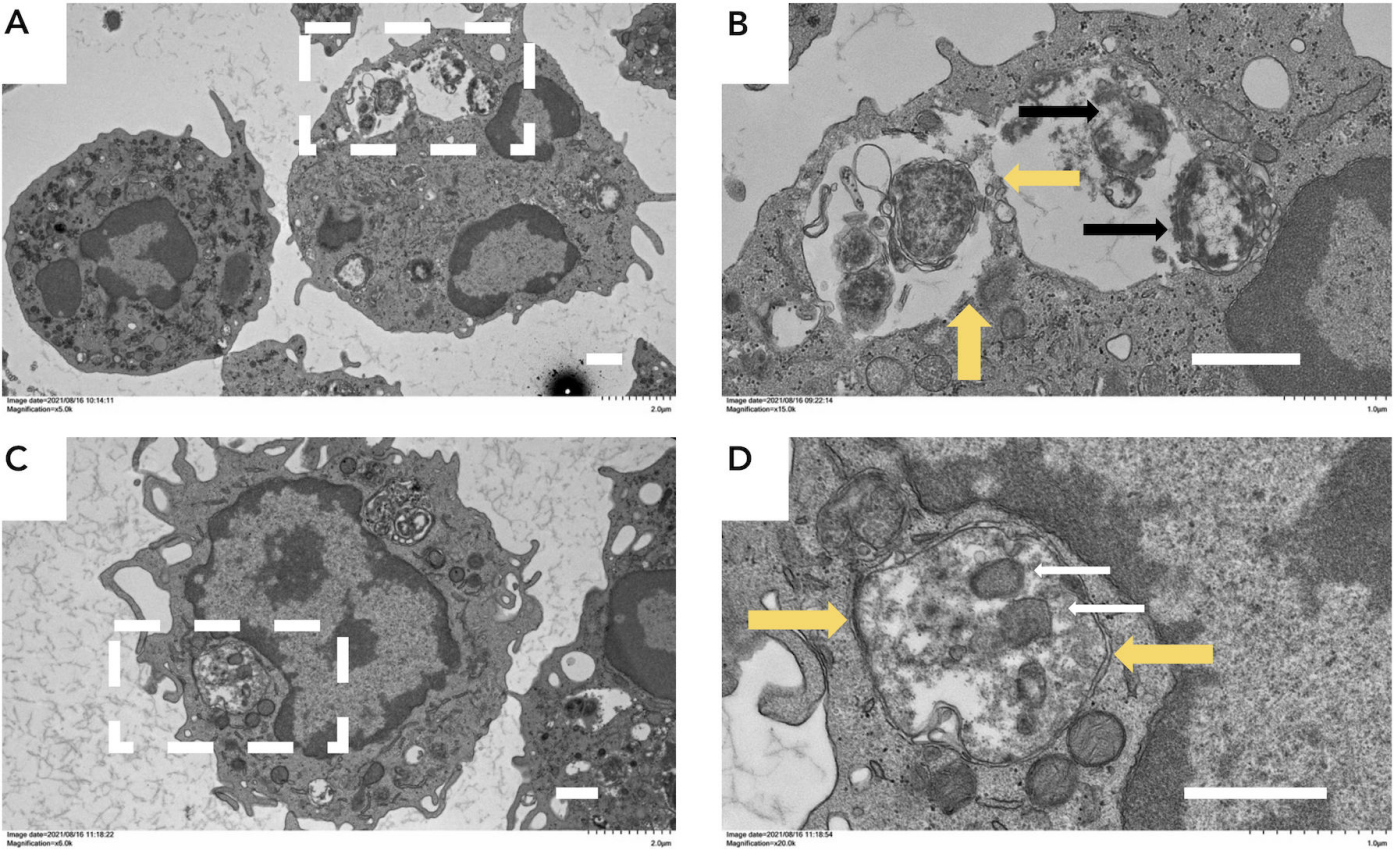
Transmission electron microscopic analysis of *N. gonorrhoeae*-infected neutrophils***.*** The figure shows transmission electron micrographs of *N. gonorrhoeae* strain 1291 (A and B) and *N. gonorrhoeae* strain 1291*pla* (C and D), taken 3 h after introduction of the respective organisms to human neutrophils in suspension. The boxed areas in panels A and C are enlarged in panels B and D. The major differences are highlighted by arrows. The large yellow arrows in panels B and D indicate the phagosomal membrane. In panel B, this membrane is disrupted, and the vacuoles appear to be fusing; whereas, in Panel D the phagosomal membrane appears to be intact. The black arrows in Panel B indicate strain 1291 disintegrating inside a phagosome. In panel D, the white arrows point to 1291*pla* within the vacuole that appears to be intact. In all panels, the scale bar represents 1.0 µm.

Previous studies have revealed that gonococci survive and replicate within neutrophils. These phagocytic cells are generally considered to play a critical role as the first line of defense against bacterial infections, albeit they appear to play a minimal role in controlling infections caused by *N. gonorrhoeae,* particularly in men. To examine a potential role for PLA in promoting the intracellular survival of gonococci, human neutrophils from healthy volunteers were infected with the *N. gonorrhoeae* strains FA1090 and FA1090*pla* for 3 h. As can be seen in Fig. 5A, with the wild-type *N. gonorrhoeae* FA1090 strain, there was an initial drop in viability that was followed by a recovery period such that, by 3 h post-infection, bacterial viability was comparable to the initial inoculum. Although the *N. gonorrhoeae* FA1090*pla* mutant also exhibited a drop in viability at 1 h post-infection, in contrast to the FA1090 parent strain, viability did not recover over the next 2 h. We repeated these studies with strains 1291 and 1291*pla* in a series of experiments using live/dead staining to enumerate viable intracellular gonococci (Fig. 5B). Neutrophils were infected with either strain 1291 or 1291*pla* and subjected to live/dead staining at 3 h post-infection. Within each strain, significantly (*P* ≤ 0.001) more 1291*pla* mutant bacteria were killed than remained viable in these experiments; whereas, significantly (*P* ≤ 0.001) more wild-type gonococci remained viable than were killed. Moreover, there was a statistically significant (*P* ≤ 0.001) difference between the viability of the 1291 parent and the 1291*pla* mutant strain (Fig. 5B). These results indicated that introduction of the *pla* mutation into the gonococcus was associated with significantly reduced survival of the organism within neutrophils. Electron micrographs of human neutrophils infected with *N. gonorrhoeae* strains 1291 and 1291*pla* for 3 h are shown in Fig. 6. As can be seen, when compared to cells infected with the 1291*pla* mutant, neutrophils infected with the 1291 parent strain have lost plasma membrane definition and have cytoplasmic disruption due to the disruption of phagolysosomes. These differences reflect the impact of the *pla* mutation, and the effect of PLA on the phagolysosome, on the survival of the gonococcus as well as the inability of neutrophils to clear a gonococcal infection.

Similar gonococcal survival studies performed using primary human cervical epithelial (Pex) cells were even more dramatic (Fig. 5C). There was no significant (*P* ≥ 0.9575) difference in the ability of the parent or 1291*pla* mutant strain to adhere to Pex cells. When compared to the parent strain, the 1291*pla* mutant exhibited a modest, but significant (*P* ≤ 0.0001), defect in its ability to invade Pex cells. However, by 3 h post-invasion, the intracellular survival of the 1291*pla* mutant within Pex cells was significantly (*P* ≤ 0.0001) reduced when compared to the parent strain. Taken together, our data indicate that the outer membrane protein, PLA, functions to enable the intracellular survival of gonococci within human neutrophils and human cervical cells.

## DISCUSSION

The rationale behind this search for a gonococcal membrane lysin was prompted by a paper studying the fate of gonococci in adherent neutrophils (11, 12). A series of electron micrographs in Fig. 3 of that paper show gonococci-infected neutrophils with large vacuoles containing numerous bacteria (11). We hypothesized that these vacuoles formed over time with gonococcal replication and with the degradation and fusion of neutrophil phagolysosomes. The figure is provided in the supplemental data as Fig. S5. Using lysis of human erythrocytes as a surrogate for the identification of a gonococcal lysin, we found that *pla* encoded a membrane-bound phospholipase A (PLA) with this capability.

Our studies show that PLA is a contact-dependent lysin, an observation with implications for gonococcal pathogenesis. PLA has been shown in other bacteria to possess similar properties (14–19, 33). Studies in *R. prowazekii* show that this pathogen has a PLA that functions in vacuolar lysis (15, 33). Similarly, in *Legionella pneumophilia*, it is demonstrated that both vacuole disruption and host cell death were largely dependent on a PLA (19). In addition to its (hemo)lytic activity, our data indicate an important role for PLA in promoting gonococcal survival during human neutrophil and cervical epithelial cell infections and, thus, the potential utility of this protein as a gonococcal vaccine antigen. With regard to *Neisseria meningitidis,* the expression of capsular polysaccharide is suggested to block PLA accessibility to bactericidal antibodies (13). Indeed, anti-PLA antibodies or immune sera are not effective in killing *N. meningitidis* in *in vitro* serum bactericidal assays (13). Whether serum bactericidal activity can serve as a correlate of protection for gonococcal disease is a subject of debate. Regardless, *N. gonorrhoeae* is an acapsular organism; thus, PLA is expected to be surface-exposed (13). The potential utility of targeting gonococcal PLA to prevent or prohibit gonorrheal disease requires further examination and will be an area of future study.

Bacteria PLAs are homodimers in which two identical peptides are non-covalently linked by a Ca^2+^ ion (32). In some cases, they are shown to reside in the outer membrane of Gram-negative bacteria. Kingma et al. have proposed that the *E. coli* outer membrane phospholipase A1 may play a role in modifying the structure of the outer membrane in response to stress (34). It is also proposed that PLA is a housekeeping protein responsible for maintaining outer membrane integrity. Recent studies indicate that phosphorylcholine is an integral component of the gonococcal membrane and, by acting on this substrate, PLA may alter the fluidity of the gonococcal membrane. The known enzymatic activity of phospholipase A is characterized as cleaving phosphatidylcholine and releasing one of the two lipids from the choline phospholipid core structure. The crystal structures of both monomeric and inhibited dimeric *E. coli* enzymes have been determined (32). This revealed that outer membrane phospholipase A1 monomers are folded into a 12-stranded antiparallel β-barrel. The active site of the *E. coli* outer membrane phospholipase A1 consists of a triad structure and has been identified as residues Ser144, His142, and neutral asparagine (Asn156) (32, 34).

With the elucidation of the gonococcal genome, the structure and location within the bacterial environment of a number of important proteins have been identified. An unexpected observation was suspicion that a PLA homolog may be in the outer membrane of the gonococcus. Our studies have confirmed this suspicion.

Herein, we initiated studies of this outer membrane phospholipase A. We found that it exhibits PLA activity in addition to functioning as a lysin resulting in beta-type hemolysis of human erythrocytes. This is the first observation of any hemolytic activity in *N. gonorrhoeae*. Bacterial hemolysins are known to be virulence factors in a number of bacterial species. Bacterial phospholipases play varied roles in human disease from triggering bacterial entry, endosomal lysis, and cytolysis to modulating the local immune response and stimulating cytokine secretion. If gonococcal PLA is regulated by changes in outer membrane integrity, as is observed for *E. coli* PLA, an interesting speculation to consider is whether the interaction with host innate immune factors within the phagolysosome (e.g., lysozyme, hypochlorous acid, superoxide, and myeloperoxidase) may initiate damage (stress) that, in turn, stimulates the increased production and release of PLA, thereby, enhancing phagolysosome membrane destruction. Minor differences in membrane composition then also could potentially provide one explanation for the modest PLA hemolytic activity (<50% of the control) that we observed in 4 of the 29 strains we tested (see Table 1), despite the apparent expression of PLA in these strains. However, whether other, strain-specific, surface factors are needed to mediate the host cell contact required for lysis to occur cannot be ruled out.

Untreated gonorrhea is not a benign disease (35). Prior to the development of antibiotics, it is estimated that 40% of all women in the US had gonorrhea at least once in their lives. In 1912, the average death rate due to gonorrhea was 32 per 100,000 per year, and the proportion of sterility due to gonorrhea ranged from 30% to 50%. Ophthalmia neonatorum was the cause of 25% of all blindness in the US, and infant mortality due to gonorrhea was 30 per 100,000. From 15% to 30% of all gynecological surgery was due to gonorrhea. For men in the US, before antibiotics, it was estimated that 60% of men had gonorrhea at least once in their lives. In the US Army, the hospital admission rate for gonorrhea was 135/1,000 personnel (35). Given the increasing problem of antibiotic resistance, these past problems could again become a reality. Thus, the need to more completely understand the pathogenesis of gonococcal infections with subsequent vaccine development is crucial.

## SUPPLEMENTAL MATERIAL

Supplemental material mbio.02425-24-s0001.pdf Table S1 and Figures S1 to S5.

ASM does not own the copyrights to Supplemental Material that may be linked to, or accessed through, an article. The authors have granted ASM a non-exclusive, world-wide license to publish the Supplemental Material files. Please contact the corresponding author directly for reuse.
