# Supplementary material for: The phospholipase A of Neisseria gonorrhoeae lyses eukaryotic membranes and is necessary for survival in neutrophils and cervical epithelial cells
Source: mBio. 2024 Sep 26;15(10):e02425-24. doi: 10.1128/mbio.02425-24 (PMC11481481; doi:10.1128/mbio.02425-24)
Supplement: Supplemental material — Table S1 and Figures S1 to S5. [file mbio.02425-24-s0001.pdf]

SUPPLEMENTAL MATERIALS:

Table S1. Percent amino acid sequence identity versus *N. gonorrhoeae pldA*

Identity (%)*	Bacterial species
29.58% - 32.68%	*Campylobacter coli*
31.47% - 35.25%	*Campylobacter jejuni*
34.81% - 39.63%	*Escherichia coli*
34.94% - 61.54%	*Pseudomonas aeruginosa*
33.78%	*Rickettsiales* sp.
31.13%	*Treponema* sp.
32.70% - 65.38%	*Vibrio vulnificus*

*BASTP search using AAW90130:phospholipase A1 from *N. gonorrhoeae* FA1090 as a query

Table S2. Primers

Primers	Sequence 5’-3’
**1492truncF**	GCCGTCTGAACGCTATATTCTTTTGACAGGACTGTTGC
**1492truncR**	TTCAGACGGCTCATCTTGCTTGATTCCGACAAAGTCCG
**NGO1492F**	ATGCCGACAATGGGGGCGGAGATGAATACACGAAATATGC
**NGO1492R**	TCAGATGCCGTCCCAGTCGTTGAACATCAACCCGATACCG
**PLA primer 1**	CGG CAC GAG TAC CGG CGT TTT AAA C
**PLA primer 2**	CCC GCG CCC CAT AAA CAC C
**PLA -414**	AGCGCACAAGCACGCAAACG
**PLA +508**	TGTCGGACAGCAGCGATTCGG
**PLA/ermC Up R**	TGCAGGCATGCAAGCTTGGCACTCGCATTGCAGCGCGGTCTCT
**PLA/ermC Down F**	GCCCTGCCACTCATCGCAGTGATCTGTTCAACGACTGGGACGGCA
**P2936**	ATGTTTGACAGCTTATCATCGATACGACCGCATCGCCGACAAAG
**P2937**	TGTGATAAACTACCGCATTAATCAGATGCCGTCCCAGTCGTTGAA

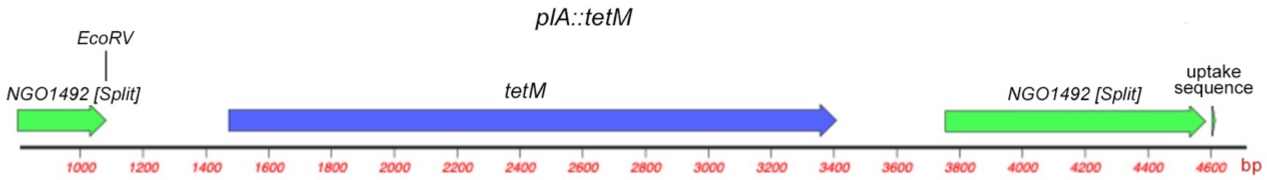

Figure S1. Schematics of pT7blue*plA::tetM* Construct. Construction of the plasmid: Thick filled arrows represent the size and orientation of the constructed gene.

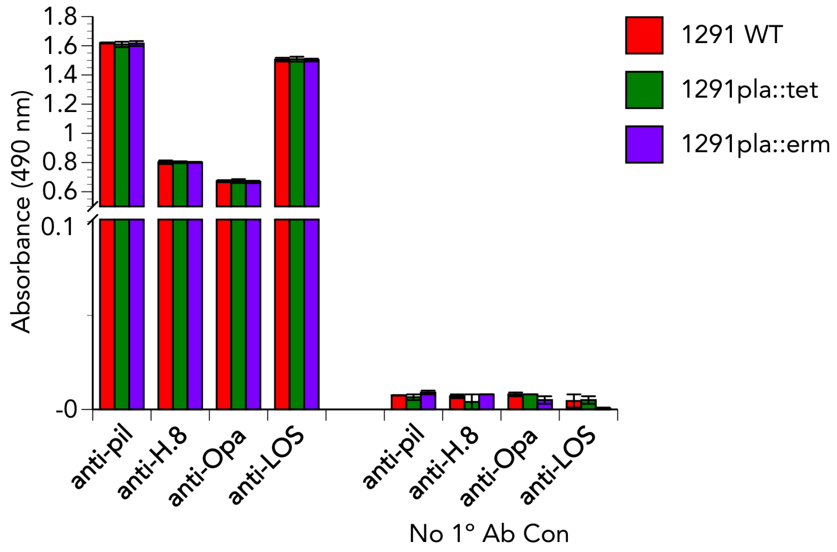

Figure S2. Expression of key surface factors by 1291 wildtype and *pla* mutant *N. gonorrhoeae.* ELISAs were performed to identify if potential differences existed in the expression of key gonococcal, phase variable, surface antigens between the 1291 parent and the 1291*pla::tet* and 1291*pla::erm* mutant strains. In brief, select wells within microtiter plates were coated with 10^7 of the noted (x-axis) gonococcal strains, after which wells were rinsed and non-specific binding sites were blocked with PBS-0.25% BSA-0.05% Tween-20. Pilin (pil), Opa, and lipooligosaccharide (LOS) then were quantitated using standard ELISA protocols and antibodies: rabbit anti-pilin, 2533; mouse anti-Opa, 4B12; and mouse-anti LOS, 6B4. The constitutively expressed H.8 antigen served as an internal control and was probed using mouse antibody 2C3. Peroxidase-conjugated, anti-rabbit or anti-mouse secondary antibodies were then used, as appropriate. Absorbance (490nm) was recorded using a Synergy HT Multi-mode microplate reader (BioTek). Blank wells, as

well as the omission of the primary antibody, served as controls for non-specific binding. Data shown were adjusted for background (blank wells) and were obtained from a single representative experiment performed in quadruplicate. Assays were performed on two separate occasions with near identical results. Statistical significance of data obtained was determined using the Student's *t*-test; no significant differences were observed (all comparisons $p \geq 0.1454$).

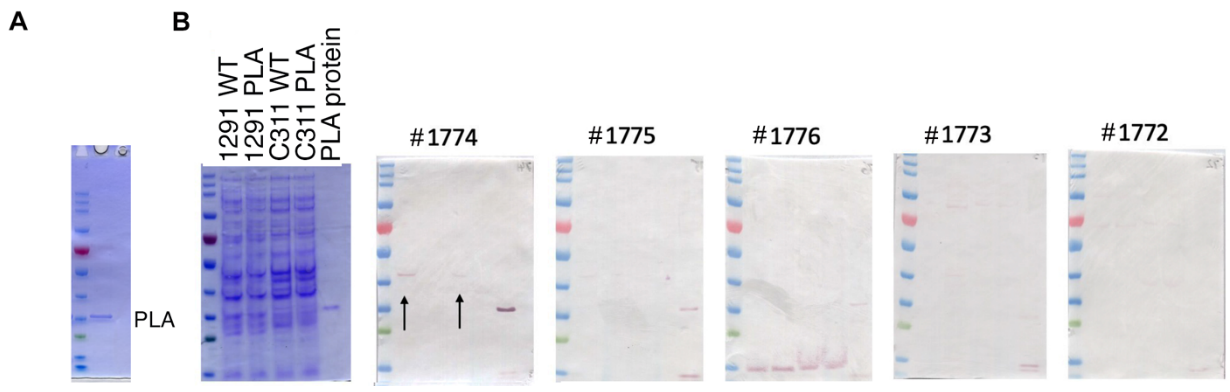

Figure S3. Analysis of anti-PLA antibody in murine test bleeds. (A) Purified N-terminus-truncated, recombinant PLA protein. (B) Western Blot analysis of mice test bleeds against PLA protein expressed in *N. gonorrhoeae* (1291) and *N. meningitidis* (C311) and recombinant PLA protein. Arrows denote reactivity of the test bleed from mouse #1774 with PLA of *N. gonorrhoeae* strain1291 and *N. meningitidis* strain C311, which is not observed in their respective *pla* mutants.

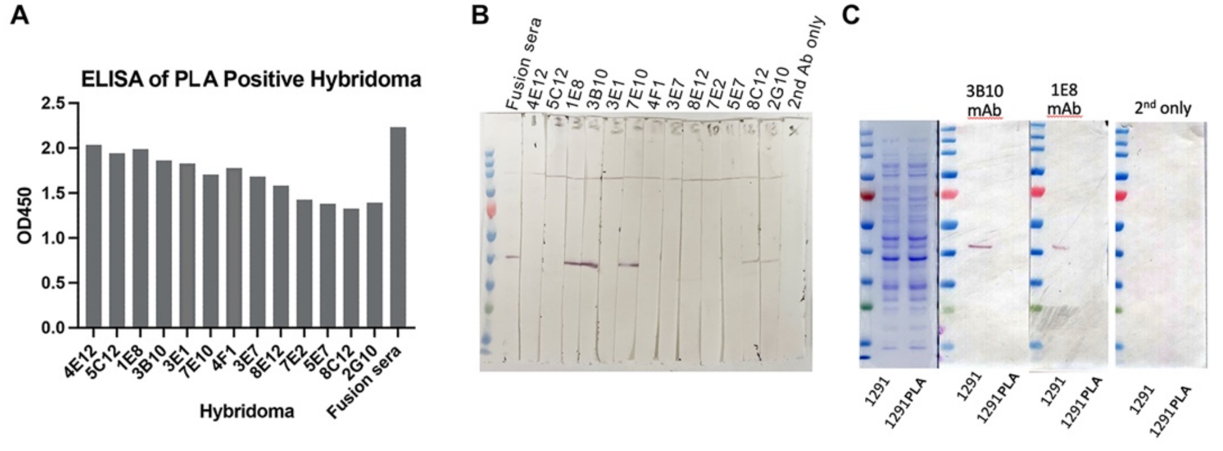

Figure S4. Verification of anti-PLA antibody specificity. (A) Microtiter plates were coated with 50 ng of recombinant PLA protein, and ELISA analysis was conducted using hybridoma culture supernatants (noted on the x-axis). (B) Western Blot analyses of hybridoma culture supernatants against *N. gonorrhoeae* (strain 1291). Hybridoma supernatants used to probe Western Blots are noted along the top edge of the membrane image. Strong reactivity was observed for mAbs 1E8, 3B10, and 7E10. (C) Western Blot analysis of purified mAb 3B10 and 1E8. Reactivity was observed for 1291 wildtype, but not 1291*pla* mutant, gonococci, demonstrating the specificity of each antibody.

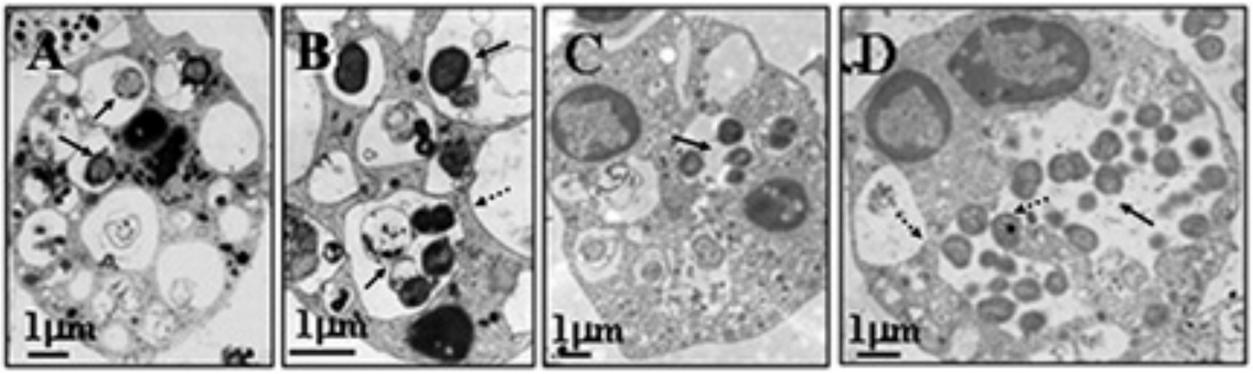

Figure S5 – Electron micrograph of human neutrophils infected with *N. gonorrhoeae* 1291 from 1 to 6 hours after infection. Figure S5 was derived from Simons *et al.* (2005) with permission from the publisher (11). (A) Gonococcal ingestion by PMNs is evident after one hour of infection. Solid arrows denote intact gonococci within phagolysosomes. Panel B shows ingestion at two hours post-infection, demonstrating individual phagolysosomes containing multiple gonococci (solid arrow) as the organism divides within the PMN. Note the phagolysosomal membranes within the PMN are intact in Panels A and B (dotted arrow). Panel C shows the infection at 4 hours. There is an increase in the number of gonococci (solid arrow) within the PMN and multiple organisms are seen within several vacuoles. Panel D shows the infection of the PMN after 6 hours. This demonstrates the complete breakdown of the PMN phagolysosomal membranes with fusion of multiple phagolysosomes (dotted arrow) and the release of large numbers of gonococci (solid arrow).
